# Exploratory analysis on the association of dietary live microbe and non-dietary prebiotic/probiotic intake with serum cotinine levels in the general adult population

**DOI:** 10.3389/fnut.2024.1405539

**Published:** 2024-05-28

**Authors:** Shanhong Lin, Ning Zhu, Yujing Zhu, Haiping Mao, Shengmin Zhang

**Affiliations:** ^1^Department of Ultrasound, The First Affiliated Hospital of Ningbo University, Ningbo, China; ^2^Department of Respiratory and Critical Care Medicine, The First Affiliated Hospital of Ningbo University, Ningbo, China; ^3^Department of Stomatology, The Affiliated Fuyang Hospital of Anhui Medical University, Fuyang, China; ^4^Department of Ultrasound, Ninghai Third Hospital, Ningbo, China

**Keywords:** dietary live microbes, probiotic/prebiotic intake, serum cotinine, NHANES, smoking

## Abstract

**Background:**

Previous research has indicated the potential involvement of the microbiota in smoking-related processes. The present study seeks to examine the relationship between dietary live microbes, as well as probiotic or prebiotic consumption, and serum cotinine levels.

**Methods:**

This study used data from the National Health and Nutrition Examination Survey 1999–2018. Dietary intake information and probiotic/prebiotic intake data was collected through self-reported questionnaires. Participants were stratified into low, medium, and high intake groups according to their consumption of foods with varying microbial content. Multiple linear models were applied to explore the relationships of dietary live microbes, probiotic or prebiotic use with the serum cotinine level.

**Results:**

A total of 42,000 eligible participants were included in the final analysis. The weighted median serum cotinine level was 0.05 (0.01, 10.90) ng/ml. Participants with low, medium, and high dietary microbe intake represented 35.4, 43.6, and 21.0% of the cohort, respectively. Furthermore, participants were stratified into three groups based on their overall consumption of foods with variable microbe contents. The association between dietary live microbe intake and serum cotinine levels remained robust across all models, with medium intake as the reference (Model 2: β = −0.14, 95% CI: −0.20, −0.07; High: β = −0.31, 95% CI: −0.39, −0.22). Moreover, both prebiotic and probiotic use exhibited an inverse relationship with serum cotinine levels (Prebiotic: β = −0.19, 95% CI: −0.37, −0.01; Probiotic: β = −0.47, 95% CI: −0.64, −0.30). Subgroup analyses revealed no discernible interactions between dietary live microbe, prebiotic, probiotic use, and serum cotinine levels.

**Conclusion:**

Our findings suggest a negative correlation between dietary live microbe intake, as well as non-dietary prebiotic/probiotic consumption, and serum cotinine levels.

## Introduction

Despite increased awareness of the adverse effects of cigarette smoking and ongoing tobacco control efforts, the global prevalence of regular smokers remains substantial, affecting 22.3% of the world’s population (1). Smoke exposure has been shown to increase susceptibility to infections caused by pathogens and exacerbate conditions such as asthma (2). The deleterious constituents of cigarette smoke, notably nicotine, are widely recognized as significant contributors to severe illnesses, prompting extensive investigation into their underlying pathological mechanisms (3). As a primary immediate metabolite of nicotine, cotinine serves as a reliable and sensitive indicator of exposure to cigarette smoke. Besides tobacco smoking, nicotine has been found in flora and some vegetables, such as tomatoes, potatoes, and peppers (4). While the nicotine content in these foods is typically low compared to tobacco products, its presence can still contribute to cotinine levels, albeit to a lesser extent (4).

Cigarette smoking and cessation can impact the gut environment, potentially inducing alterations in the commensal microbial community, including shifts in gut microbiota balance (5). Numerous studies have indicated that smoking may alter the composition of the periodontal, upper esophageal, and gastric, as well as respiratory microbiomes (6–8). An intriguing question arises regarding the potential influence of gut microbiota on smoking behaviors. Recent research suggests that disruptions in gut microbiota, termed dysbiosis, are implicated in various chronic human diseases (9). For instance, dysbiosis may contribute to the development of hypertension (10). Short-chain fatty acids (SCFAs), such as propionate, produced by gut bacteria from dietary fiber, have been found to mitigate organ damage associated with hypertension (10). Additionally, the antibiotic azithromycin has been observed to modulate gut microbiota, leading to reduced airway inflammation in individuals with allergic asthma (11). However, the involvement of microbiota in smoking-related chronic diseases, including chronic obstructive pulmonary disease, remains unclear.

Probiotics and prebiotics, commonly integrated into dietary supplements, which probiotics are living microorganisms characterized by their capacity to bestow health benefits upon the host and prebiotics are non-living substances that promote the growth and activity of beneficial bacteria in the gut. Numerous studies have underscored the therapeutic potential of probiotics in managing asthma and respiratory infections (12, 13). Probiotics, as living microorganisms, confer significant health advantages to the host when administered in sufficient quantities (14). Administration of probiotics has been shown to augment the development of immune and metabolic conditions such as obesity, diabetes, and inflammatory bowel disease (14, 15). Prebiotics, on the other hand, are non-digestible components in food that exert positive effects by selectively fostering the growth and activity of specific bacteria in the colon, ultimately enhancing individuals’ health (16). Moreover, prebiotics exhibit beneficial effects on conditions such as diarrhea, obesity, type II diabetes, and colorectal cancer (17).

Currently, substantial research attention has been devoted to the microbiota-gut-brain axis, which posits that alterations in gut microbiota may influence brain function (18). Notably, neurological functions of the brain have been found to be intricately linked to cigarette smoke. Specifically, two brain regions—the orbitofrontal cortex and the prefrontal cortex—have been identified as interacting to either activate or suppress nicotine cravings (19, 20). Furthermore, animal studies have directly illustrated that manipulation of the gut microbiome can modulate behaviors related to rewards and stress associated with substance abuse, including tobacco (21, 22). Hence, the microbiota may indeed play a role in the process of smoking, and consumption of live microbes could represent a potential avenue for addressing cigarette use. However, the precise relationships between dietary live microbes and smoking behaviors remain unclear. Our study seeks to investigate the association of dietary live microbes, probiotic or prebiotic usage, with serum cotinine levels, leveraging data from a large population obtained from the National Health and Nutrition Examination Survey (NHANES).

## Materials and methods

### Study population

The NHANES is an ongoing program conducted by the National Center for Health Statistics within the Centers for Disease Control and Prevention, administered through a stratified, multistage probability sampling design.^1^ All data and information from NHANES are publicly accessible. Trained interviewers collected participant data via administered standardized questionnaires in participants’ homes. Written informed consent was obtained from all individuals, and all research protocols were approved by the National Center for Health Statistics’ ethical review board. Further detailed information can be found on the NHANES website.

**Figure 1:**
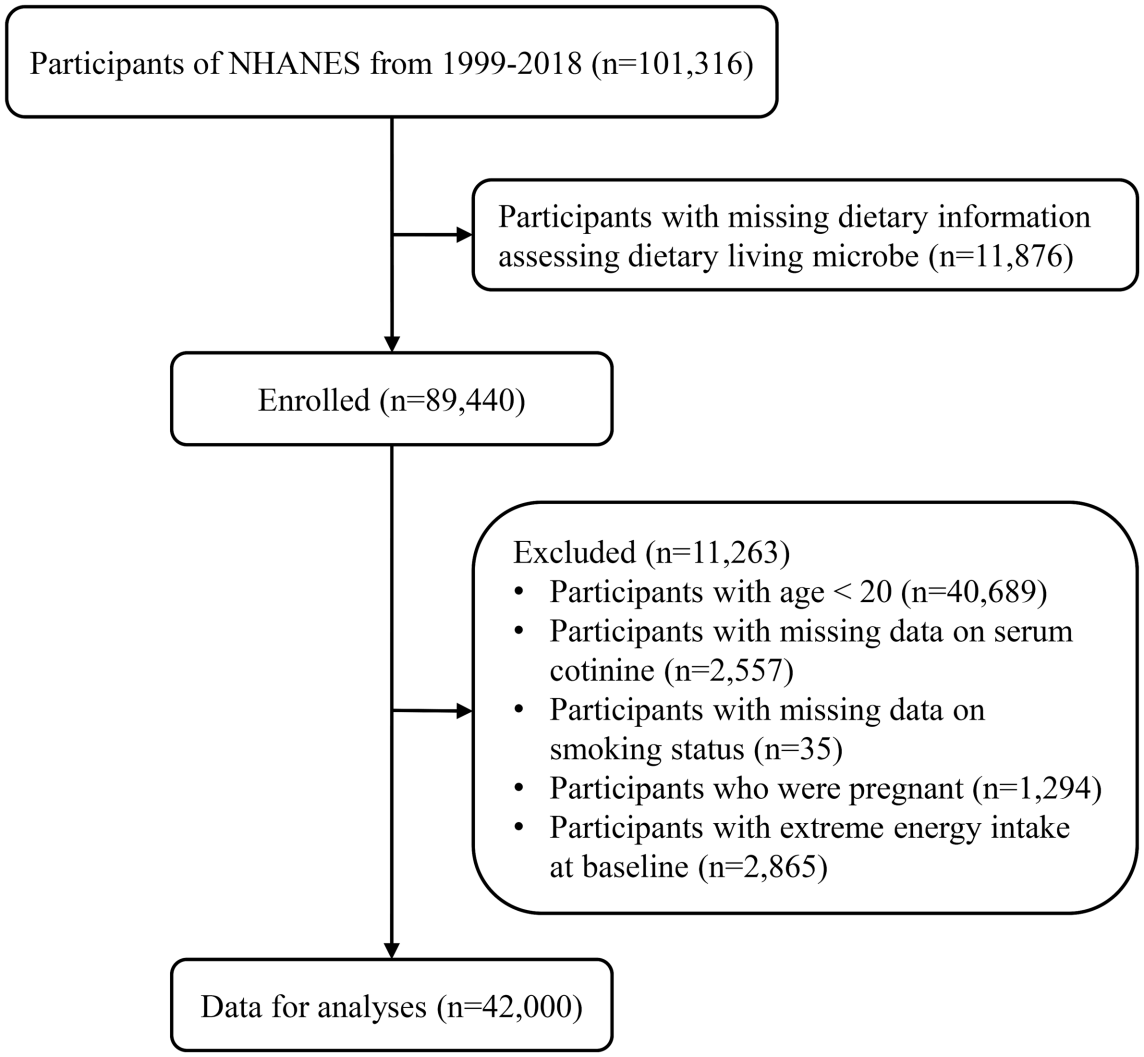
Flowchart of the study participants.

We collected data and characteristics of 101,316 participants from NHANES between 1999 and 2018, following NHANES sample weight guidelines.^2^ Participants with missing data assessing dietary live microbe intake (*n* = 11,876) were excluded. Additionally, individuals aged less than 20 (*n* = 40,689), those with missing serum cotinine data (*n* = 2,557), missing smoking status data (*n* = 35), pregnant individuals (*n* = 1,294), and those with extreme energy intake at baseline (*n* = 2,865) were excluded. In total, 42,000 eligible participants were included in the final analyses. Detailed information on the sample selection procedure is displayed in Figure 1.

### Assessment of live microbe intake from foods and non-dietary prebiotic/probiotic intake

The estimation of dietary live microbe intake was established using a methodology developed by professional experts based on a total of 9,388 participant food codes in 48 subgroups within the NHANES program (23). Utilizing estimated levels of live microbial content, including bacteria and fungi, foods were classified into three groups based on the quantity of live microorganisms per gram of food: low (Lo, <10^4^ CFU/g in foods), medium (Med, 10^4^–10^7^ CFU/g in foods), and high (Hi, >10^7^ CFU/g in foods). The designations of Lo, Med, and Hi were selected to represent the expected quantities of viable microbes in foods. The Hi classification primarily consists of fermented items, such as yogurt and fermented kimchi (23). The Med classification comprises fresh fruits and vegetables typically consumed without peeling (23). The Lo levels could represent the numbers of microbes in pasteurized foods (23). Additionally, a fourth category, named MedHi, was established, encompassing individuals who consume foods from the Med, Hi, or both the Medium and High categories. When both Med and Hi foods were simultaneously analyzed as separate terms in the same model, their coefficients were found to be similar. This finding supports a simpler model where the exposure combines Med and Hi foods into a single variable termed MedHi (23). Non-dietary probiotic and prebiotic intake data were collected from the Food Frequency Questionnaire (FFQ) and Dietary Supplement Use 30-Day (DSQ) using detailed text-mining for key phrases.

Participants were stratified into three distinct groups based on their overall consumption of foods with varying contents of live microbes: low (participants exclusively consumed foods classified as Low in microbial content), moderate (participants consumed any foods classified as Medium but not High), and high (participants consumed any foods classified as High). Additionally, participants were categorized into three groups based on their consumption of MedHi foods to quantify the ingestion of live microbes: Group 1 (G1) comprised consumers with no intake of any MedHi food; Group 2 (G2) consisted of individuals with intake of MedHi food above zero but below the median level for consumers; and Group 3 (G3) encompassed participants with intake of MedHi food above the median level for consumers.

### Assessment of serum cotinine

Serum cotinine measurements were obtained via venipuncture and analyzed utilizing an isotope-dilution high-performance liquid chromatography/atmospheric pressure chemical ionization tandem mass spectrometric (ID HPLC-APCI MS/MS) method. In brief, serum samples were fortified with methyl-D3-cotinine and methyl-D3-hydroxycotinine as internal standards. Following basification, the samples were applied to a supported liquid extraction (SLE) plate. Analytes were extracted using an isopropanol/methylene chloride mixture, and the resulting organic extract was concentrated before injection onto a C18 HPLC column. The mobile phase used for chromatographic separation consisted of solvent A (6 mM ammonium acetate in water/methanol) and solvent B (methanol). The chromatographic run was performed using gradient elution with a flow rate of 0.5 mL/min, column temperature of 30°C, and a gradient profile of 10–90% solvent B over 5 min. The total run time was 10 min, with an injection volume of 10 microliters. APCI-MS/MS monitored the eluent from these injections. Cotinine was quantified using the m/z 80 product ion from the m/z 177 quasi-molecular ion. Additional ions for the internal standards and confirmation were also monitored. Analyte concentrations were determined from the area ratios of native-to-labeled compounds in the sample, compared to a standard curve. Further details on specific laboratory procedures can be found on the website.^3^

### Assessment of other covariates

We assessed the covariates as follows: age (20–39 years, 40–59 years, or ≥ 60 years), sex, race (non-Hispanic White, non-Hispanic Black, or other races), marital status (married/living with partner, or single/divorced/widowed), education level (below high school, high school, above high school), family poverty-to-income ratio (PIR), smoking status, drinking status, body mass index (BMI, <25.0 kg/m^2^, 25.0–29.9 kg/m^2^, or > 29.9 kg/m^2^), physical activity, healthy eating index (HEI), Charlson comorbidity index.

PIR served as an indicator of family income levels (24), with three categories established: ≤1.0, 1.1–3.0, >3.0. Smoking status was categorized into three groups: never smoker (individuals reporting having smoked fewer than 100 cigarettes throughout their lifetime), former smoker (those reporting having smoked more than 100 cigarettes but subsequently quit), current smoker (actively engaged in smoking) (25). Drinking status was divided into three groups: nondrinker, low-to-moderate drinker (consuming alcohol at a rate of less than 2 drinks per day for men and less than 1 drink per day for women), heavy drinker (exceeding 2 drinks per day for men and 1 or more drinks per day for women) (25). Physical activity was categorized based on metabolic equivalent (MET) levels, including three categories: inactive (no leisure-time physical activity), insufficiently active (moderate activity 1–5 times per week with MET ranging from 3 to 6, or vigorous activity 1–3 times per week with MET greater than 6), and active (engagement in physical activity beyond the levels described in the aforementioned categories) (25). The Healthy Eating Index (HEI) is a metric devised by the United States Department of Agriculture (USDA) to evaluate the overall nutritional quality of an individual’s or population’s diet (26). It furnishes a comprehensive score derived from multiple facets of dietary intake, indicative of adherence to fundamental dietary guidelines. HEI computation was predicated on the dietary intakes recorded on the initial day for each participant, adhering to HEI scoring standards outlined on the National Cancer Institute’s Epidemiology and Genomics Research Program website.^4^ The Charlson comorbidity index was utilized to quantify participants’ holistic health status, entailing the summation of scores attributed to various chronic conditions (27).

### Statistical analyses

Adhering to the analytical guidelines delineated by the National Center for Health Statistics (NCHS), our analysis incorporated primary sampling units, sample weights, and strata to yield dependable national estimates. Data analyses were conducted employing the “survey” package in R to conduct weighted analyses. Participant characteristics were presented as means with standard errors (SEs) for continuous variables and as numbers with weighted percentages for categorical variables. Group-wise comparisons for continuous variables were executed utilizing weighted t-tests or one-way ANOVA, while categorical variables were compared among groups using weighted chi-square tests.

Multiple linear regression models were deployed to evaluate the relationship between dietary live microbe and non-dietary prebiotic/probiotic intake with serum cotinine levels among American adults. β coefficients and 95% confidence intervals were employed to assess these associations. In regression models utilizing dietary live microbe as a continuous exposure, the reported regression coefficients denote the adjusted mean difference in the outcome per 100-unit increase in the exposure. We formulated two models: Model 1, adjusted for age (20–39, 40–59, or ≥ 60), sex (male or female), race/ethnicity (non-Hispanic White, non-Hispanic Black, or other race), marital status (married/living with partner, or single/divorced/widowed), education level (below high school, high school, or above high school), family poverty-to-income ratio (≤1.0, 1.1–3.0, or > 3.0), drinking status (nondrinker, former drinker, or current drinker), BMI (<25.0, 25.0–29.9, or > 29.9), physical activity (inactive, insufficiently active, or active), Healthy Eating Index (HEI, in quartiles), and Charlson comorbidity index (continuous); Model 2, adjusted as Model 1 with the inclusion of smoking status (never smoker, former smoker, or current smoker).

Subgroup analyses, stratified by age, sex, race, marital status, educational level, family poverty-to-income ratio, smoking status, drinking status, physical activity, BMI, HEI, and Charlson comorbidity index, were conducted using stratified multivariate regression analysis. Additionally, we constructed forest plots depicting β coefficients and confidence intervals in different subgroups to facilitate visualization of the relationships between non-dietary prebiotic and probiotic intake and serum cotinine levels, respectively. All analyses were performed using R (version 4.3.2), and statistical significance was defined as *p* < 0.05.

## Results

### Baseline characteristics according to MedHi category

Table 1 presented baseline characteristics for 42,000 participants who consumed MedHi foods. Among them, 14,852 individuals were categorized in the low microbe consumption group, 18,302 individuals in the moderate microbe consumption group, and 8,846 individuals in the high microbe consumption group. Of the total participants, 13,252 (35.70%) were aged 20–39 years, 13,756 (38.29%) were aged 40–59 years, and 14,992 (26.01%) were aged ≥60 years. There were 21,629 (52.31%) females and 20,371 (47.69%) males. The weighted median of serum cotinine was 0.05 ng/mL. Participants in the high MedHi category were more likely to be middle-aged (40–59 years old), female, non-Hispanic White, single/divorced/widowed, never smokers, low-to-moderate drinkers, engaged in active physical activity, with higher family PIR, higher education level, lower Charlson comorbidity index, and lower serum cotinine level (all *p* < 0.001, except for Charlson comorbidity index where *p* = 0.02). Additionally, Supplementary Table S1 outlines the distribution of dietary live microbe intake among adults in the NHANES 1999–2018. Supplementary Table S2 provides a comprehensive overview of the sociodemographic and health status characteristics of adult participants from the NHANES 1999–2018, stratified by serum cotinine levels (<10 ng/mL, or ≥ 10 ng/mL).

**Table 1:** Baseline characteristics of the adult participants by category of MedHi in NHANES 1999–2018.

Characteristics	Total	Category of MedHi*			*p-*value
		Low	Medium	High	
Participants, n	42,000	14,852	18,302	8,846	
Age, years					<0.001
20–39	13,252(35.70)	5,077(39.88)	5,210(32.51)	2,965(35.63)	
40–59	13,756(38.29)	4,760(37.39)	6,015(38.64)	2,981(38.88)	
≥60	14,992(26.01)	5,015(22.73)	7,077(28.86)	2,900(25.49)	
Sex, %					<0.001
Female	21,629(52.31)	7,101(48.15)	9,529(53.21)	4,999(56.15)	
Male	20,371(47.69)	7,751(51.85)	8,773(46.79)	3,847(43.85)	
Race/ethnicity, %					<0.001
Non-Hispanic White	19,237(69.84)	6,040(64.05)	8,290(69.44)	4,907(77.91)	
Non-Hispanic Black	8,316(10.22)	4,091(15.42)	3,138(9.04)	1,087(5.55)	
Other race	14,447(19.93)	4,721(20.53)	6,874(21.52)	2,852(16.54)	
Marital status, %					<0.001
Married/living with partner	16,543(35.51)	6,564(40.60)	6,793(33.67)	3,186(32.04)	
Single/divorced/widowed	25,457(64.49)	8,288(59.40)	11,509(66.33)	5,660(67.96)	
Education level, %					<0.001
Below high school	11,057(16.58)	4,552(21.57)	4,958(16.60)	1,547(10.18)	
High school	9,706(23.92)	3,895(28.44)	4,078(23.03)	1733(19.60)	
Above high school	21,237(59.50)	6,405(49.99)	9,266(60.37)	5,566(70.22)	
Family PIR, %					<0.001
≤1.0	8,399(13.73)	3,659(18.30)	3,406(12.58)	1,334(9.77)	
1.1–3.0	17,702(35.96)	6,706(40.90)	7,722(35.54)	3,274(30.33)	
>3.0	15,899(50.31)	4,487(40.80)	7,174(51.88)	4,238(59.89)	
Smoking status, %					<0.001
Never smoker	22,745(53.98)	7,439(49.12)	10,187(55.36)	5,119(57.92)	
Former smoker	10,705(25.25)	3,451(22.49)	4,959(26.88)	2,295(26.09)	
Current smoker	8,550(20.77)	3,962(28.39)	3,156(17.76)	1,432(15.99)	
Drinking status, %					<0.001
Nondrinker	9,718(18.84)	3,642(20.41)	4,344(19.72)	1732(15.36)	
Low-to-moderate drinker	28,978(71.89)	10,014(70.55)	12,608(71.43)	6,356(74.36)	
Heavy drinker	3,304(9.27)	1,196(9.04)	1,350(8.84)	758(10.28)	
Body mass index, %					<0.001
<25.0 kg/m^2^	12,079(30.52)	4,149(28.89)	5,237(30.75)	2,693(32.22)	
25.0–29.9 kg/m^2^	14,313(33.48)	4,825(31.62)	6,396(34.20)	3,092(34.68)	
>29.9 kg/m^2^	15,608(36.00)	5,878(39.49)	6,669(35.06)	3,061(33.10)	
Physical activity, %					<0.001
Inactive	11,467(21.94)	4,605(25.72)	4,860(21.38)	2002(18.04)	
Insufficiently active	15,660(40.37)	5,344(39.81)	6,943(41.10)	3,373(39.90)	
Active	14,873(37.68)	4,903(34.47)	6,499(37.53)	3,471(42.06)	
HEI	49.90(40.72,59.74)	44.99(36.75,53.65)	52.24(43.05,61.90)	52.90(43.26,62.72)	<0.001
Charlson comorbidity index	0.88(0.01)	0.87(0.02)	0.91(0.02)	0.84(0.02)	0.020
Serum cotinine, ng/mL	0.05(0.01,10.90)	0.12(0.02,132.00)	0.04(0.01,1.20)	0.03(0.01,0.39)	<0.001

PIR, poverty income ratio; HEI, Healthy Eating Index. Normally distributed continuous variables are described as means ± SEs, and continuous variables without a normal distribution are presented as medians (interquartile ranges). Categorical variables are presented as numbers (percentages). Sampling weights were applied for calculation of demographic descriptive statistics; N reflect the study sample while percentages reflect the survey-weighted data. *Participants were also classified into three different groups considering the general intake of foods with varying contents of microbe: low (all foods consumed were Low); moderate (any foods consumed were Medium but not High); and high (any foods consumed were High).

### Associations of dietary live microbe and non-dietary prebiotic/probiotic intake with serum cotinine levels

The results of linear regression analysis examining the associations of dietary live microbe and non-dietary prebiotic/probiotic intake with serum cotinine levels are presented in Table 2. Dietary live microbe intake exhibited a negative correlation with serum cotinine levels. Specifically, each 100-unit increase in MedHi foods was associated with average reductions in serum cotinine levels of −0.4 (95% CI: −0.43, −0.37; *p* < 0.001) ng/mL in crude analysis, −0.17 (95% CI: −0.19, −0.14; *p* < 0.001) ng/mL in model 1, and − 0.07 (95% CI: −0.09, −0.06; *p* < 0.001) ng/mL in model 2. Furthermore, when participants were stratified into three groups based on their general intake of foods with varying microbial contents, the association between dietary live microbe and serum cotinine levels remained significant across all models (with the Low category as reference; Model 2: Medium: β = −0.14, 95% CI: −0.20, −0.07; High: β = −0.31, 95% CI: −0.39, −0.22, *p* < 0.001). Similarly, categorization of participants into three groups based on MedHi consumption to quantify live microbe ingestion yielded significant results (with G1 as reference; Model 2: G2: β = −0.13, 95% CI: −0.20, −0.06; G3: β = −0.28, 95% CI: −0.36, −0.20, *p* < 0.001). Regarding prebiotic use outcomes, estimated effects demonstrated a negative association with serum cotinine levels across all models (Crude: β = −1.05, 95% CI: −1.36, −0.75; Model 1: β = −0.51, 95% CI: −0.80, −0.21; Model 2: β = −0.19, 95% CI: −0.37, −0.01). Similarly, probiotic use exhibited an inverse relationship with serum cotinine levels (Crude: β = −1.43, 95% CI: −1.69, −1.17; Model 1: β = −0.72, 95% CI: −0.99, −0.44; Model 2: β = −0.47, 95% CI: −0.64, −0.30).

**Table 2:** Linear regression analysis of dietary live microbe and non-dietary prebiotic/probiotic intake with serum cotinine levels among adults in NHANES 1999–2018.

	Crude		Model 1		Model 2	
	β (95% CI)	*P* value	β (95% CI)	*P* value	β (95% CI)	*P* value
Per 100 unit increase	−0.4 (−0.43, −0.37)	<0.001	−0.17 (−0.19, −0.14)	<0.001	−0.07 (−0.09, −0.06)	<0.001
Category of MedHi*						
Low	0 [Reference]		0 [Reference]		0 [Reference]	
Medium	−1.22 (−1.33, −1.11)	<0.001	−0.48 (−0.59, −0.38)	<0.001	−0.14 (−0.20, −0.07)	<0.001
High	−1.6 (−1.74, −1.45)	<0.001	−0.74 (−0.87, −0.62)	<0.001	−0.31 (−0.39, −0.22)	<0.001
Category of MedHi†						
G1	0 [Reference]		0 [Reference]		0 [Reference]	
G2	−0.87 (−0.99, −0.74)	<0.001	−0.41 (−0.52, −0.30)	<0.001	−0.13 (−0.20, −0.06)	<0.001
G3	−1.83 (−1.96, −1.71)	<0.001	−0.78 (−0.90, −0.67)	<0.001	−0.28 (−0.36, −0.20)	<0.001
Prebiotic use						
No	0 [Reference]		0 [Reference]		0 [Reference]	
Yes	−1.05 (−1.36, −0.75)	<0.001	−0.51 (−0.80, −0.21)	0.001	−0.19 (−0.37, −0.01)	0.036
Probiotic use						
No	0 [Reference]		0 [Reference]		0 [Reference]	
Yes	−1.43 (−1.69, −1.17)	<0.001	−0.72 (−0.99, −0.44)	<0.001	−0.47 (−0.64, −0.30)	<0.001

Model 1 was adjusted for age (20–39, 40–59, or ≥ 60), sex (male or female), race/ethnicity (Non-Hispanic White, Non-Hispanic Black or Other), marital status (married/living with partner, or single/divorced/widowed), education level (below high school, high school, or above high school), family PIR (≤1.0, 1.1–3.0, or > 3.0), drinking status (nondrinker, former drinker, or current drinker), BMI (<25.0, 25.0–29.9, or > 29.9), physical activity (inactive, insufficiently active, or active), HEI (in quartiles), and Charlson comorbidity index (continuous); Model 2 was adjusted as model 1 plus smoking status (never smoker, former smoker, or current smoker). *Participants were also classified into three different groups considering the general intake of foods with varying contents of microbe: low (all foods consumed were Low); moderate (any foods consumed were Medium but not High); and high (any foods consumed were High). ^†^Participants were categorized into three groups based on the MedHi consumption to quantify the ingestion of live microbes: G1, consumers without intakes of any MedHi food; G2, those with intakes of MedHi food above zero but below the median level for consumers; G3, those with intakes of MedHi food above the median level for consumers.

### Subgroup analysis and interaction analysis

**Figure 2:**
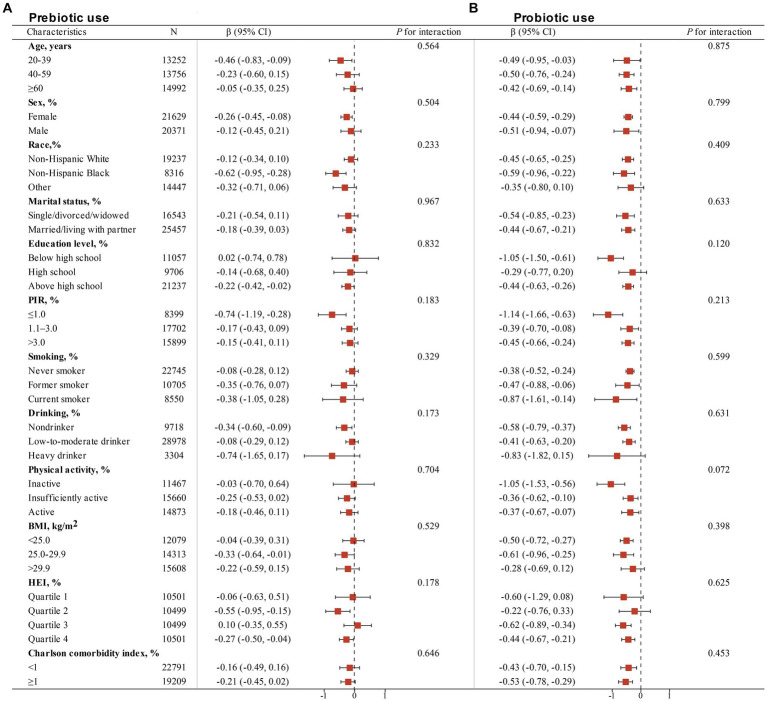
Stratified analyses of the associations of non-dietary prebiotic **(A)** and probiotic **(B)** intake with serum cotinine levels among adults in NHANES 1999–2018. Analyses were adjusted for covariates age (20–39, 40–59, or ≥ 60), sex (male or female), race/ethnicity (Non-Hispanic White, Non-Hispanic Black or Other), marital status (married/living with partner, or single/divorced/widowed), education level (below high school, high school, or above high school), family PIR (≤1.0, 1.1–3.0, or > 3.0), drinking status (nondrinker, former drinker, or current drinker), BMI (<25.0, 25.0–29.9, or > 29.9), physical activity (inactive, insufficiently active, or active), HEI (in quartiles), and Charlson comorbidity index (continuous), and smoking status (never smoker, former smoker, or current smoker) when they were not the strata variables.

Table 3 presents the associations of dietary live microbe intake within different subgroups stratified by various covariates with serum cotinine levels. An interaction test, stratified by age, sex, race, marital status, education level, PIR, smoking status, drinking status, physical activity, BMI, HEI, and Charlson comorbidity index, was conducted. A significant interaction between race (*P* for interaction = 0.007), PIR (*P* for interaction = 0.037), smoking status (*P* for interaction = 0.007) and serum cotinine levels. However, no statistically significant interaction between dietary live microbe intake and serum cotinine levels was observed in other subgroups. This indicates that the association remained consistent across different demographic and health-related variables, including age, sex, marital status, education level, alcohol consumption, physical activity, BMI, HEI, and Charlson Comorbidity Index (all *P* for interaction >0.05). Figure 2 illustrates the stratified analyses of the associations between prebiotic and probiotic use and serum cotinine levels through a forest plot. No discernible interaction was found between prebiotic and probiotic use and serum cotinine levels in these subgroup results.

**Table 3:** Stratified analyses of the associations between dietary live microbe intake and serum cotinine levels among adults in NHANES 1999–2018.

Subgroups	N	Category of MedHi*			*P*_interaction_
		Low	Medium	High	
Age, years					0.790
20–39	13,252	0 [Reference]	−0.19 (−0.30, −0.09)	−0.34 (−0.48, −0.20)	
40–59	13,756	0 [Reference]	−0.07 (−0.20, 0.06)	−0.22 (−0.37, −0.07)	
≥ 60	14,992	0 [Reference]	−0.13 (−0.23, −0.02)	−0.36 (−0.48, −0.25)	
Sex, %					0.256
Female	21,629	0 [Reference]	−0.16 (−0.24, −0.09)	−0.26 (−0.36, −0.16)	
Male	20,371	0 [Reference]	−0.09 (−0.21, 0.02)	−0.35 (−0.49, −0.21)	
Race, %					0.007
Non-Hispanic White	19,237	0 [Reference]	−0.11 (−0.20, −0.02)	−0.29 (−0.39, −0.19)	
Non-Hispanic Black	8,316	0 [Reference]	0.02 (−0.09, 0.13)	−0.27 (−0.44, −0.10)	
Other	14,447	0 [Reference]	−0.16 (−0.28, −0.04)	−0.26 (−0.42, −0.11)	
Marital status, %					0.815
Married/living with partner	16,543	0 [Reference]	−0.11 (−0.23, 0.00)	−0.31 (−0.45, −0.17)	
Single/divorced/widowed	25,457	0 [Reference]	−0.15 (−0.24, −0.06)	−0.30 (−0.41, −0.20)	
Education level, %					0.372
Below high school	11,057	0 [Reference]	−0.22 (−0.35, −0.09)	−0.34 (−0.52, −0.16)	
High school	9,706	0 [Reference]	−0.13 (−0.29, 0.02)	−0.27 (−0.44, −0.10)	
Above high school	21,237	0 [Reference]	−0.08 (−0.17, 0.01)	−0.28 (−0.38, −0.17)	
Family PIR, %					0.037
≤1.0	8,399	0 [Reference]	−0.27 (−0.39, −0.15)	−0.36 (−0.56, −0.16)	
1.1–3.0	17,702	0 [Reference]	−0.18 (−0.28, −0.08)	−0.25 (−0.39, −0.11)	
>3.0	15,899	0 [Reference]	−0.05 (−0.17, 0.07)	−0.3 (−0.42, −0.18)	
Smoking status, %					0.007
Never smoker	22,745	0 [Reference]	−0.14 (−0.23, −0.04)	−0.25 (−0.37, −0.13)	
Former smoker	10,705	0 [Reference]	−0.12 (−0.31, 0.07)	−0.43 (−0.65, −0.21)	
Current smoker	8,550	0 [Reference]	−0.13 (−0.21, −0.05)	−0.19 (−0.32, −0.06)	
Drinking status, %					0.424
Nondrinker	9,718	0 [Reference]	−0.18 (−0.30, −0.06)	−0.32 (−0.49, −0.16)	
Low-to-moderate drinker	28,978	0 [Reference]	−0.09 (−0.16, −0.01)	−0.26 (−0.35, −0.17)	
Heavy drinker	3,304	0 [Reference]	−0.41 (−0.68, −0.14)	−0.56 (−0.85, −0.27)	
Physical activity, %					0.419
Inactive	11,467	0 [Reference]	−0.12 (−0.25, 0.00)	−0.21 (−0.37, −0.06)	
Insufficiently active	15,660	0 [Reference]	−0.15 (−0.26, −0.03)	−0.30 (−0.44, −0.17)	
Active	14,873	0 [Reference]	−0.13 (−0.25, −0.01)	−0.36 (−0.51, −0.20)	
Body mass index, %					0.723
<25.0 kg/m^2^	12,079	0 [Reference]	−0.17 (−0.29, −0.06)	−0.30 (−0.44, −0.17)	
25.0–29.9 kg/m^2^	14,313	0 [Reference]	−0.13 (−0.26, 0.00)	−0.31 (−0.45, −0.17)	
>29.9 kg/m^2^	15,608	0 [Reference]	−0.11 (−0.22, 0.00)	−0.29 (−0.44, −0.14)	
HEI, %					0.091
Quartile 1	10,501	0 [Reference]	−0.01 (−0.15, 0.13)	−0.15 (−0.31, 0.00)	
Quartile 2	10,499	0 [Reference]	−0.16 (−0.29, −0.03)	−0.40 (−0.54, −0.25)	
Quartile 3	10,499	0 [Reference]	−0.20 (−0.33, −0.08)	−0.43 (−0.59, −0.26)	
Quartile 4	10,501	0 [Reference]	−0.19 (−0.34, −0.05)	−0.3 (−0.47, −0.13)	
Charlson comorbidity index, %					0.424
<1	22,791	0 [Reference]	−0.12 (−0.22, −0.03)	−0.30 (−0.41, −0.20)	
≥1	19,209	0 [Reference]	−0.16 (−0.26, −0.07)	−0.33 (−0.46, −0.20)	

Analyses were adjusted for covariates age (20–39, 40–59, or ≥ 60), sex (male or female), race/ethnicity (Non-Hispanic White, Non-Hispanic Black or Other), marital status (married/living with partner, or single/divorced/widowed), education level (below high school, high school, or above high school), family PIR (≤1.0, 1.1–3.0, or > 3.0), drinking status (nondrinker, former drinker, or current drinker), BMI (<25.0, 25.0–29.9, or > 29.9), physical activity (inactive, insufficiently active, or active), HEI (in quartiles), and Charlson comorbidity index (continuous), and smoking status (never smoker, former smoker, or current smoker) when they were not the strata variables. *Participants were also classified into three different groups considering the general intake of foods with varying contents of microbe: low (all foods consumed were Low); moderate (any foods consumed were Medium but not High); and high (any foods consumed were High).

### Sensitivity analysis

To ensure the robustness of the findings, a series of sensitivity analyses were conducted. Supplementary Table S3 presents the results of a sensitivity analysis conducted to examine the association between dietary live microbe intake and serum cotinine levels categorized as <10 ng/mL or ≥ 10 ng/mL among adults participating in NHANES 1999–2018. Consistent with the linear regression analysis, the results of logistic regression indicated that increased intake of dietary live microbe and non-dietary prebiotic/probiotic intake were associated with reduced odds of having serum cotinine levels ≥10 ng/mL (Supplementary Table S3). This association remained statistically significant across all models. Supplementary Table S4 presents the results of stratified analyses investigating the associations between dietary live microbe intake and serum cotinine levels (<10 ng/mL or ≥ 10 ng/mL), stratified by various demographic and health-related factors. The findings suggest that the association between dietary live microbe intake and serum cotinine levels was robust and not significantly modified by demographic or health-related factors examined in this analysis.

Supplementary Tables S5, S6 present the results of linear regression analyses investigating the relationship between serum cotinine levels and dietary live microbe intake, as well as non-dietary prebiotic/probiotic intake among adults participating in NHANES 1999–2018. Despite this difference in categorization of dietary live microbe intakes in Supplementary Tables S5, S6, the results consistently demonstrated an inverse association between dietary live microbe intake and serum cotinine levels. Similarly, individuals who reported prebiotic or probiotic use exhibited lower serum cotinine levels compared to those who did not use prebiotics or probiotics.

## Discussion

This study investigated the association between dietary intake of live microorganisms and non-dietary consumption of prebiotics/probiotics with serum cotinine levels among adults. Our findings revealed that the consumption of foods containing live microbes was associated with lower serum cotinine levels. This negative association persisted across different levels of MedHi categorization (G1–G3) in Table 2. Similarly, the use of probiotic and prebiotic supplements demonstrated similar results. Subgroup analyses and interaction assessments consistently indicated the persistence of this negative association across various demographic and health-related factors (Table 3). To the best of our knowledge, this study represents the first comprehensive investigation into the association between dietary intake of live microorganisms and non-dietary consumption of prebiotics/probiotics with serum cotinine levels.

The consumption of live and safe microbes has been advocated as a recommendation due to their association with health benefits (28). Dietary intake of live microbes has been demonstrated to reduce markers of inflammation, enhance immunity, modulate stress responses, and mitigate the risk of several chronic diseases (28, 29). One plausible explanatory mechanism for these findings revolves around the interconnectedness of food, the gut, and overall health (30). An expanding body of evidence, albeit partly indirect, suggests potential biological rationales for the impact of commensal gut microbiota, particularly probiotics such as *Bifidobacterium*, on smoking behavior. The vagus nerve is believed to serve as a prominent modulatory and constitutive communication pathway linking intestinal bacteria to the brain (31). Through the vagus nerve, *Bifidobacterium longum* has been observed to transmit signals to the brain, leading to increased dopamine secretion (31). Additionally, the influence of the intestinal microbiome on smoking behavior may be mediated through neurotransmitters. Some reports indicate that *Bifidobacterium* contributes to enhanced serotonin (5-HT) biosynthesis in colonic enterochromaffin cells (32). This process is achieved by promoting the activity of the CGA/ADRα2A pathway and modulating the TRP/TPH-OR pathways (33). Given that 5-HT has been identified as a therapeutic target for addiction to various substances, including alcohol, cocaine, and drugs, it is conceivable that targeting serotonin may also offer therapeutic potential for smoking addiction (34). The observed impact of *Bifidobacterium* on smoking behavior may be attributed to the close association of its metabolites, such as short-chain fatty acids like acetate, or components like peptidoglycan, with the central nervous system (CNS) (35, 36). Short-chain fatty acids may play a significant role in microglial function and brain physiology (37). While these studies provide some evidence for the association between gut microbiota and smoking behavior, the underlying mechanisms remain incompletely understood. The gap between serum cotinine levels and comprehensive smoking activity has not been investigated at the population level. Future studies should focus on elucidating specialized mechanisms and strengthening observational evidence in this domain.

Moreover, a previous meta-analysis has indicated a significant enhancement in host oxidative stress homeostasis through the substantial improvement of glutathione (GSH) levels following probiotic or prebiotic consumption (38). Additionally, lactic acid bacteria have been shown to ameliorate oxidative imbalance among antioxidant factors by regulating AhR and Nrf2 gene expression in bronchial epithelial cells exposed to cigarette smoke (39). A recent animal study demonstrated that the probiotic *Bifidobacterium longum* subsp. *longum* alleviates lung inflammation induced by cigarette smoke in mice, as evidenced by a reduction in the count of immune cells in bronchoalveolar lavage fluid (BALF) and lung parenchyma (40). The administration of *Bifidobacterium* probiotics has been associated with a decreased occurrence of respiratory infections in humans (41, 42). The reduced expression of adhesion molecules Icam1 and Vcam1 is linked to the mitigation of immune cell adhesion and migration from the bloodstream to lung tissue, highlighting the anti-inflammatory effects of *B. longum* subsp. *longum*. The aforementioned evidence suggests that probiotics may have the potential to alleviate the burden of smoking-related chronic diseases by addressing their pathogenic mechanisms. Prebiotics are non-digestible fibers that selectively stimulate the growth and activity of beneficial bacteria in the gut (43). Prebiotics can alter the composition of the gut microbiota, favoring the growth of certain beneficial bacteria over others (44). In turn, these beneficial bacteria can influence various metabolic processes in the body, including those related to xenobiotic metabolism, such as nicotine (45, 46). Prebiotics have been shown to have anti-inflammatory and antioxidant effects in some studies. A study explored the influence of prebiotics in bread on antioxidative status and antioxidative ability in smokers and non-smokers (47). They found that consuming prebiotic bread could decrease the levels of stress biomarkers (47). The potential relationship between prebiotics and cotinine levels has not been extensively studied, more mechanisms need to be explored.

The decrease of cotinine in plasma typically correlates with a reduction in tobacco consumption. Cotinine is a metabolite of nicotine, serving as a reliable and objective measure of nicotine exposure (48). Cotinine has a longer half-life than nicotine and remains detectable in bodily fluids such as blood, urine, and saliva for a longer duration, making it an excellent biomarker for assessing recent tobacco exposure (48). Cigarette smoking has been demonstrated to induce alterations in the composition of the gut microbiota (49). Compared to non-smokers, current smokers exhibit a decreased Firmicutes/Bacteroidetes (F/B) ratio and a lower relative abundance of the phyla Firmicutes and Proteobacteria (49). Several studies have suggested that the gut microbiota may play a role in both the development and potential treatment of smoking-induced diseases. The gut microbiota may have the potential to metabolize cotinine (50). Some research indicated that certain bacterial species can metabolize nicotine and its major metabolites into other compounds (50). Prebiotics and probiotics represent promising targets for treatment in chronic diseases caused by cigarette smoking, such as chronic obstructive pulmonary disease (COPD). Previous studies have indicated that the administration of probiotic *B. longum* subsp. *longum* mitigates lung inflammation induced by cigarette smoke in mice (40). Administration of probiotics, such as *L. rhamnosus* and *P. goldsteinii*, may attenuate symptoms in tobacco smoking-induced COPD by reducing the inflammatory response (51, 52). Prebiotics, as non-digestible food components that promote the growth and activity of beneficial bacteria in the colon, have also shown promise in promoting overall health (16). Feeding mice a high-fiber diet rich in cellulose or pectin for 3 weeks has demonstrated a protective effect against the progression of emphysema induced by cigarette smoking (53). This evidence suggests that utilizing probiotics tailored to the composition of the gut microbiota could serve as a promising therapeutic approach for ameliorating symptoms associated with chronic diseases caused by tobacco smoking.

Our study has some advantages. Our research represents the inaugural investigation into the correlation between dietary live microbes and serum cotinine levels on a large scale within a nationally representative program. This pioneering effort lays the groundwork for future studies in this area. Moreover, by utilizing data from a nationally representative program, our study offers insights that are likely to be generalizable to the broader population. This enhances the external validity of our findings and contributes to a better understanding of the relationship between dietary live microbes and serum cotinine levels at a national level. Furthermore, we comprehensively assessed dietary live microbe intake and serum cotinine levels, providing a detailed examination of these variables within the context of smoking behavior. This thorough approach strengthens the robustness of our analysis and allows for a more nuanced interpretation of the results.

However, several limitations of our research should be mentioned. First of all, the cross-sectional nature of our study limits our ability to establish causal relationships between dietary live microbe intake, prebiotic/probiotic consumption, and serum cotinine levels. Future longitudinal studies are warranted to elucidate the directionality of these associations over time. Moreover, the reliance on self-reported dietary live microbe and prebiotic/probiotic intake data introduces the potential for memory bias and reporting inaccuracies. Participants may not accurately recall their dietary habits, leading to measurement errors and potential misclassification of exposure levels. Thirdly, while we made efforts to estimate dietary live microbe intake, the precision of these measurements may be compromised due to inherent limitations in quantifying microbial content in food. Variations in food processing, storage conditions, and microbial composition introduce uncertainty into these estimates. Further, our findings may not be directly applicable to populations outside of the study’s geographic region or cultural context. The classification of food and the prevalence of live microbe intake can vary significantly across different regions and populations, limiting the generalizability of our results.

## Conclusion

Our study, the first of its kind to investigate the relationship between dietary live microbes and serum cotinine levels on a large, nationally representative scale, yields significant insights into the interplay between microbiota and exposure to cigarette smoke. We found a consistent negative association between the consumption of live microbes, probiotics, or prebiotics and serum cotinine levels, suggesting a potential protective effect against the harmful effects of smoking. Future studies should employ longitudinal designs to better elucidate causality and explore the potential therapeutic implications of modulating the gut microbiota in individuals exposed to cigarette smoke.

## Data availability statement

The datasets presented in this study can be found in online repositories. The names of the repository/repositories and accession number(s) can be found in the article/Supplementary material.

## Ethics statement

The studies involving humans were approved by the CDC/NCHS Research Ethics Review Board from NAHENS. The studies were conducted in accordance with the local legislation and institutional requirements. The participants provided their written informed consent to participate in this study. The animal study was approved by the CDC/NCHS Research Ethics Review Board from NHANES. The study was conducted in accordance with the local legislation and institutional requirements.

## Author contributions

SL: Conceptualization, Data curation, Formal analysis, Investigation, Software, Writing – original draft, Writing – review & editing. NZ: Conceptualization, Data curation, Formal analysis, Investigation, Methodology, Writing – original draft, Writing – review & editing. YZ: Data curation, Methodology, Project administration, Resources, Writing – review & editing. HM: Data curation, Formal analysis, Methodology, Software, Writing – review & editing. SZ: Conceptualization, Data curation, Project administration, Resources, Supervision, Validation, Visualization, Writing – review & editing.

## Footnotes

^1^https://www.cdc.gov/nchs/nhanes/index.htm

^2^https://wwwn.cdc.gov/nchs/nhanes/analyticguidelines.aspx

^3^https://wwwn.cdc.gov/Nchs/Nhanes/2013-2014/COT_H.htm

^4^https://epi.grants.cancer.gov/hei/hei-scoring-method.html
