# Supplementary material for: Exploratory analysis on the association of dietary live microbe and non-dietary prebiotic/probiotic intake with serum cotinine levels in the general adult population
Source: Front Nutr. 2024 May 28;11:1405539. doi: 10.3389/fnut.2024.1405539 (PMC11165358; doi:10.3389/fnut.2024.1405539)
Supplement: Supplementary file 1 [file Table_1.docx]

**Supplementary Materials**

**Exploratory analysis on the Association of Dietary Live Microbe and Nondietary Prebiotic/Probiotic Intake with Serum Cotinine Levels in the General Adult Population**

**Table S1.** Distributions of dietary live microbe intake (g/day) among adults in NHANES 1999–2018 ^*^.

| Food category ^†^ | N ^‡^ | Mean | 50^th^ percentile | 75^th^ percentile | 95^th^ percentile |
| --- | --- | --- | --- | --- | --- |
| Lo | 42,000 | 3049.17±17.24 | 2782.15±17.13 | 3760.91±22.72 | 5767.50±43.34 |
| Med | 24,919 | 102.95±1.79 | 38.00±1.78 | 153.75±2.85 | 402.33±5.92 |
| Hi | 8,846 | 20.00±0.51 | 0.00±1.21 | 2.35±1.65 | 142.50±4.53 |
| MedHi | 27,148 | 122.95±2.09 | 56.70±1.46 | 182.19±2.03 | 458.19±7.15 |

*Combined data from the NHANES 1999-2018 for adult participants (n = 42,000). Data presented as gram of intake per day ± standard error.

†Utilizing estimated levels of live microbial content, including bacteria and fungi, foods were classified into three groups based on the quantity of live microorganisms per gram of food: low (Lo, <104 CFU/g in foods), medium (Med, 104–107 CFU/g in foods), and high (Hi, >107 CFU/g in foods). Additionally, a fourth category, named MedHi, was established, encompassing individuals who consume foods from the Med, Hi, or both the Medium and High categories.

‡ In total of 6617 participants consumed both Med and Hi foods; hence, the sum of the number of consumers of Med and Hi foods exceeds the total of Med or Hi foods. When referring to dietary intake. MedHi refers to foods that were categorized as either having medium or high microbial content; 14852 study subjects consumed neither Med nor Hi foods.

**Table S2.** Survey-weighted, sociodemographic and health status characteristics of adult NHANES 1999–2018 participants with available data.

| Characteristics | Total (n=42000) | Serum cotinine, ng/mL | | *P* value |
| --- | --- | --- | --- | --- |
|  |  | <10 ng/mL (n=31806) | ≥10 ng/mL (n=10194) |  |
| Age, years |  |  |  | <0.001 |
| 20-39 | 13252(35.70) | 9288(33.16) | 3964(43.26) |  |
| 40-59 | 13756(38.29) | 9950(37.39) | 3806(40.98) |  |
| ≥ 60 | 14992(26.01) | 12568(29.45) | 2424(15.76) |  |
| Sex, % |  |  |  | <0.001 |
| Female | 21629(52.31) | 17461(55.57) | 4168(42.58) |  |
| Male | 20371(47.69) | 14345(44.43) | 6026(57.42) |  |
| Race/ethnicity, % |  |  |  | <0.001 |
| Non-Hispanic White | 19237(69.84) | 14104(69.24) | 5133(71.64) |  |
| Non-Hispanic Black | 8316(10.22) | 5667(9.22) | 2649(13.23) |  |
| Other race | 14447(19.93) | 12035(21.54) | 2412(15.13) |  |
| Marital status, % |  |  |  | <0.001 |
| Married/living with partner | 16543(35.51) | 11728(32.90) | 4815(43.28) |  |
| Single/divorced/widowed | 25457(64.49) | 20078(67.10) | 5379(56.72) |  |
| Education level, % |  |  |  | <0.001 |
| Below high school | 11057(16.58) | 7848(14.35) | 3209(23.26) |  |
| High school | 9706(23.92) | 6715(21.11) | 2991(32.28) |  |
| Above high school | 21237(59.50) | 17243(64.54) | 3994(44.46) |  |
| Family PIR, % |  |  |  | <0.001 |
| ≤1.0 | 8399(13.73) | 5409(11.11) | 2990(21.54) |  |
| 1.1–3.0 | 17702(35.96) | 13245(34.51) | 4457(40.29) |  |
| >3.0 | 15899(50.31) | 13152(54.38) | 2747(38.18) |  |
| Smoking status, % |  |  |  | <0.001 |
| Never smoker | 22745(53.98) | 21715(68.90) | 1030(9.49) |  |
| Former smoker | 10705(25.25) | 9534(29.41) | 1171(12.84) |  |
| Current smoker | 8550(20.77) | 557(1.69) | 7993(77.67) |  |
| Drinking status, % |  |  |  | <0.001 |
| Nondrinker | 9718(18.84) | 8541(21.90) | 1177(9.71) |  |
| Low-to-moderate drinker | 28978(71.89) | 21506(71.22) | 7472(73.90) |  |
| Heavy drinker | 3304(9.27) | 1759(6.88) | 1545(16.40) |  |
| Body mass index, % |  |  |  | <0.001 |
| <25.0 kg/m^2^ | 12079(30.52) | 8564(28.94) | 3515(35.21) |  |
| 25.0-29.9 kg/m^2^ | 14313(33.48) | 11057(34.12) | 3256(31.59) |  |
| >29.9 kg/m^2^ | 15608(36.00) | 12185(36.94) | 3423(33.20) |  |
| Physical activity, % |  |  |  | 0.096 |
| Inactive | 11467(21.94) | 8750(21.75) | 2717(22.52) |  |
| Insufficiently active | 15660(40.37) | 12021(40.79) | 3639(39.12) |  |
| Active | 14873(37.68) | 11035(37.46) | 3838(38.36) |  |
| HEI | 49.90(40.72,59.74) | 51.69(42.24,61.60) | 45.12(37.22,53.66) | <0.001 |
| Charlson comorbidity index | 0.88(0.01) | 0.90(0.01) | 0.82(0.02) | <0.001 |
| Serum cotinine, ng/mL | 0.05(0.01,10.90) | 0.03(0.01, 0.07) | 210.00(113.00,313.00) | <0.001 |
| Category of MedHi* |  |  |  | <0.001 |
| Low | 14852(32.50) | 10126(28.65) | 4726(43.97) |  |
| Medium | 18302(42.08) | 14504(43.89) | 3798(36.70) |  |
| High | 8846(25.42) | 7176(27.46) | 1670(19.32) |  |
| Category of MedHi† |  |  |  | <0.001 |
| G1 | 14852(32.50) | 10126(28.65) | 4726(43.97) |  |
| G2 | 13574(32.90) | 10272(32.73) | 3302(33.39) |  |
| G3 | 13574(34.60) | 11408(38.61) | 2166(22.64) |  |

Abbreviations: PIR, poverty income ratio; HEI, Healthy Eating Index. Normally distributed continuous variables are described as means ± SEs, and continuous variables without a normal distribution are presented as medians [interquartile ranges]. Categorical variables are presented as numbers (percentages). Sampling weights were applied for calculation of demographic descriptive statistics; N reflect the study sample while percentages reflect the survey-weighted data.

*Participants were also classified into three different groups considering the general intake of foods with varying contents of microbe: low (all foods consumed were Low); moderate (any foods consumed were Medium but not High); and high (any foods consumed were High).

†Participants were categorized into three groups based on the MedHi consumption of quantify the ingestion of live microbes: G1, consumers without intakes of any MedHi food; G2, those with intakes of MedHi food above zero but below the median level for consumers; G3, those with intakes of MedHi food above the median level for consumers.

**Table S3.** Logistic regression analysis of dietary live microbe and nondietary prebiotic/probiotic intake with serum cotinine levels (<10ng/mL, or ≥10 ng/mL) among adults in NHANES 1999–2018.

|  | Crude | |  | Model 1 | |  | Model 2 | |
| --- | --- | --- | --- | --- | --- | --- | --- | --- |
|  | OR (95% CI) | *P* value |  | OR (95% CI) | *P* value |  | OR (95% CI) | *P* value |
| Per 100 unit increase | 0.76 (0.74-0.78) | <0.001 |  | 0.87 (0.85-0.89) | <0.001 |  | 0.90 (0.86-0.94) | <0.001 |
| Category of MedHi* | |  |  |  |  |  |  |  |
| Low | 1 [Reference] |  |  | 1 [Reference] |  |  | 1 [Reference] |  |
| Medium | 0.54 (0.51-0.58) | <0.001 |  | 0.75 (0.70-0.80) | <0.001 |  | 0.85 (0.77-0.94) | 0.003 |
| High | 0.46 (0.42-0.50) | <0.001 |  | 0.64 (0.59-0.70) | <0.001 |  | 0.70 (0.61-0.82) | <0.001 |
| Category of MedHi† | |  |  |  |  |  |  |  |
| G1 | 1 [Reference] |  |  | 1 [Reference] |  |  | 1 [Reference] |  |
| G2 | 0.66 (0.62-0.71) | <0.001 |  | 0.80 (0.74-0.85) | <0.001 |  | 0.87 (0.79-0.96) | 0.006 |
| G3 | 0.38 (0.36-0.41) | <0.001 |  | 0.60 (0.56-0.66) | <0.001 |  | 0.71 (0.62-0.82) | <0.001 |
| Prebiotic use |  |  |  |  |  |  |  |  |
| No | 1 [Reference] |  |  | 1 [Reference] |  |  | 1 [Reference] |  |
| Yes | 0.47 (0.35-0.65) | <0.001 |  | 0.61 (0.43-0.85) | 0.004 |  | 0.72 (0.58-0.99) | 0.042 |
| Probiotic use |  |  |  |  |  |  |  |  |
| No | 1 [Reference] |  |  | 1 [Reference] |  |  | 1 [Reference] |  |
| Yes | 0.39 (0.28-0.53) | <0.001 |  | 0.55 (0.38-0.79) | 0.002 |  | 0.50 (0.27-0.92) | 0.027 |

Model 1 was adjusted for age (20-39, 40-59, or ≥60), sex (male or female), race/ethnicity (Non-Hispanic White, Non-Hispanic Black or Other), marital status (married/living with partner, or single/divorced/widowed), education level (below high school, high school, or above high school), family PIR (≤1.0, 1.1–3.0, or >3.0), drinking status (nondrinker, former drinker, or current drinker), BMI (<25.0, 25.0-29.9, or >29.9), physical activity (inactive, insufficiently active, or active), HEI (in quartiles), and Charlson comorbidity index (continuous); Model 2 was adjusted as model 1 plus smoking status (never smoker, former smoker, or current smoker).

* Participants were also classified into three different groups considering the general intake of foods with varying contents of microbe: low (all foods consumed were Low); moderate (any foods consumed were Medium but not High); and high (any foods consumed were High).

† Participants were categorized into three groups based on the MedHi consumption of quantify the ingestion of live microbes: G1, consumers without intakes of any MedHi food; G2, those with intakes of MedHi food above zero but below the median level for consumers; G3, those with intakes of MedHi food above the median level for consumers.

**Table S4.** Stratified analyses of the associations between dietary live microbe intake and serum cotinine levels (<10ng/mL, or ≥10 ng/mL) among adults in NHANES 1999–2018.

| Subgroups | N | Category of MedHi* | | | *P*_interaction_ |
| --- | --- | --- | --- | --- | --- |
|  |  | Low | Medium | High |  |
| Age, years |  |  |  |  | 0.632 |
| 20-39 | 13252 | 1 [Reference] | 0.86 (0.73-1.02) | 0.68 (0.56-0.82) |  |
| 40-59 | 13756 | 1 [Reference] | 0.90 (0.72-1.12) | 0.81 (0.62-1.07) |  |
| ≥ 60 | 14992 | 1 [Reference] | 0.76 (0.58-0.99) | 0.54 (0.38-0.77) |  |
| Sex, % |  |  |  |  | 0.324 |
| Female | 21629 | 1 [Reference] | 0.76 (0.62-0.94) | 0.65 (0.49-0.88) |  |
| Male | 20371 | 1 [Reference] | 0.90 (0.79-1.03) | 0.72 (0.60-0.86) |  |
| Race, % |  |  |  |  | 0.134 |
| Non-Hispanic White | 19237 | 1 [Reference] | 0.84 (0.73-0.98) | 0.68 (0.56-0.82) |  |
| Non-Hispanic Black | 8316 | 1 [Reference] | 1.09 (0.91-1.30) | 0.81 (0.60-1.09) |  |
| Other | 14447 | 1 [Reference] | 0.82 (0.66-1.02) | 0.77 (0.57-1.03) |  |
| Marital status, % |  |  |  |  | 0.537 |
| Married/living with partner | 16543 | 1 [Reference] | 0.87 (0.71-1.05) | 0.64 (0.50-0.83) |  |
| Single/divorced/widowed | 25457 | 1 [Reference] | 0.85 (0.74-0.97) | 0.74 (0.61-0.89) |  |
| Education level, % |  |  |  |  | 0.647 |
| Below high school | 11057 | 1 [Reference] | 0.78 (0.63-0.98) | 0.68 (0.50-0.93) |  |
| High school | 9706 | 1 [Reference] | 0.90 (0.73-1.11) | 0.78 (0.57-1.06) |  |
| Above high school | 21237 | 1 [Reference] | 0.88 (0.74-1.04) | 0.69 (0.55-0.86) |  |
| Family PIR, % |  |  |  |  | 0.377 |
| ≤1.0 | 8399 | 1 [Reference] | 0.77 (0.61-0.98) | 0.66 (0.48-0.92) |  |
| 1.1–3.0 | 17702 | 1 [Reference] | 0.85 (0.72-1.01) | 0.81 (0.64-1.03) |  |
| >3.0 | 15899 | 1 [Reference] | 0.88 (0.72-1.07) | 0.65 (0.50-0.84) |  |
| Smoking status, % |  |  |  |  | 0.721 |
| Never smoker | 22745 | 1 [Reference] | 0.84 (0.70-1.01) | 0.79 (0.60-1.04) |  |
| Former smoker | 10705 | 1 [Reference] | 0.91 (0.75-1.10) | 0.66 (0.52-0.85) |  |
| Current smoker | 8550 | 1 [Reference] | 0.77 (0.60-1.00) | 0.63 (0.46-0.87) |  |
| Drinking status, % |  |  |  |  | 0.774 |
| Nondrinker | 9718 | 1 [Reference] | 0.73 (0.52-1.01) | 0.50 (0.29-0.88) |  |
| Low-to-moderate drinker | 28978 | 1 [Reference] | 0.89 (0.79-1.00) | 0.75 (0.64-0.88) |  |
| Heavy drinker | 3304 | 1 [Reference] | 0.75 (0.53-1.06) | 0.58 (0.39-0.86) |  |
| Physical activity, % |  |  |  |  | 0.387 |
| Inactive | 11467 | 1 [Reference] | 0.80 (0.62-1.02) | 0.85 (0.60-1.20) |  |
| Insufficiently active | 15660 | 1 [Reference] | 0.90 (0.74-1.09) | 0.66 (0.50-0.88) |  |
| Active | 14873 | 1 [Reference] | 0.84 (0.69-1.03) | 0.67 (0.52-0.87) |  |
| Body mass index, % |  |  |  |  | 0.505 |
| <25.0 kg/m^2^ | 12079 | 1 [Reference] | 0.80 (0.65-1.00) | 0.64 (0.48-0.86) |  |
| 25.0-29.9 kg/m^2^ | 14313 | 1 [Reference] | 0.83 (0.68-1.01) | 0.66 (0.52-0.84) |  |
| >29.9 kg/m^2^ | 15608 | 1 [Reference] | 0.91 (0.75-1.09) | 0.79 (0.62-1.02) |  |
| HEI, % |  |  |  |  | 0.186 |
| Quartile 1 | 10501 | 1 [Reference] | 1.03 (0.84-1.26) | 0.86 (0.68-1.08) |  |
| Quartile 2 | 10499 | 1 [Reference] | 0.80 (0.65-0.98) | 0.56 (0.42-0.74) |  |
| Quartile 3 | 10499 | 1 [Reference] | 0.79 (0.63-0.99) | 0.59 (0.43-0.83) |  |
| Quartile 4 | 10501 | 1 [Reference] | 0.73 (0.54-0.99) | 0.75 (0.50-1.12) |  |
| Charlson comorbidity index, % |  |  |  |  | 0.746 |
| <1 | 22791 | 1 [Reference] | 0.89 (0.77-1.02) | 0.73 (0.61-0.88) |  |
| ≥1 | 19209 | 1 [Reference] | 0.80 (0.66-0.96) | 0.65 (0.50-0.85) |  |

Analyses were adjusted for covariates age (20-39, 40-59, or ≥60), sex (male or female), race/ethnicity (Non-Hispanic White, Non-Hispanic Black or Other), marital status (married/living with partner, or single/divorced/widowed), education level (below high school, high school, or above high school), family PIR (≤1.0, 1.1–3.0, or >3.0), drinking status (nondrinker, former drinker, or current drinker), BMI (<25.0, 25.0-29.9, or >29.9), physical activity (inactive, insufficiently active, or active), HEI (in quartiles), and Charlson comorbidity index (continuous), and smoking status (never smoker, former smoker, or current smoker) when they were not the strata variables.

* Participants were also classified into three different groups considering the general intake of foods with varying contents of microbe: low (all foods consumed were Low); moderate (any foods consumed were Medium but not High); and high (any foods consumed were High).

**Table S5.** Linear regression analysis of serum cotinine levels according to mutual adjustment of dietary live microbe and nondietary prebiotic/probiotic intake among adults in NHANES 1999–2018.

|  | Crude | |  | Model 1 | |  | Model 2 | |
| --- | --- | --- | --- | --- | --- | --- | --- | --- |
|  | β (95% CI) | *P* value |  | β (95% CI) | *P* value |  | β (95% CI) | *P* value |
| Category of MedHi* | |  |  |  |  |  |  |  |
| Low | 0 [Reference] |  |  | 0 [Reference] |  |  | 0 [Reference] |  |
| Medium | -1.03 (-1.18, -0.88) | <0.001 |  | -0.37 (-0.51, -0.22) | <0.001 |  | -0.04 (-0.13, 0.04) | 0.300 |
| High | -1.29 (-1.45, -1.13) | <0.001 |  | -0.62 (-0.77, -0.46) | <0.001 |  | -0.20 (-0.29, -0.11) | <0.001 |
| Prebiotic use |  |  |  |  |  |  |  |  |
| No | 0 [Reference] |  |  | 0 [Reference] |  |  | 0 [Reference] |  |
| Yes | -0.70 (-1.02, -0.38) | <0.001 |  | -0.34 (-0.67, -0.01) | 0.041 |  | -0.07 (-0.26, 0.11) | 0.442 |
| Probiotic use |  |  |  |  |  |  |  |  |
| No | 0 [Reference] |  |  | 0 [Reference] |  |  | 0 [Reference] |  |
| Yes | -1.09 (-1.39, -0.79) | <0.001 |  | -0.59 (-0.91, -0.28) | <0.001 |  | -0.44 (-0.62, -0.26) | <0.001 |

Model 1 was adjusted for age (20-39, 40-59, or ≥60), sex (male or female), race/ethnicity (Non-Hispanic White, Non-Hispanic Black or Other), marital status (married/living with partner, or single/divorced/widowed), education level (below high school, high school, or above high school), family PIR (≤1.0, 1.1–3.0, or >3.0), drinking status (nondrinker, former drinker, or current drinker), BMI (<25.0, 25.0-29.9, or >29.9), physical activity (inactive, insufficiently active, or active), HEI (in quartiles), and Charlson comorbidity index (continuous); Model 2 was adjusted as model 1 plus smoking status (never smoker, former smoker, or current smoker).

* Participants were also classified into three different groups considering the general intake of foods with varying contents of microbe: low (all foods consumed were Low); moderate (any foods consumed were Medium but not High); and high (any foods consumed were High).

**Table S6.** Linear regression analysis of serum cotinine levels according to mutual adjustment of dietary live microbe and nondietary prebiotic/probiotic intake among adults in NHANES 1999–2018.

|  | Crude | |  | Model 1 | |  | Model 2 | |
| --- | --- | --- | --- | --- | --- | --- | --- | --- |
|  | β (95% CI) | *P* value |  | β (95% CI) | *P* value |  | β (95% CI) | *P* value |
| Category of MedHi† | |  |  |  |  |  |  |  |
| G1 | 0 [Reference] |  |  | 0 [Reference] |  |  | 0 [Reference] |  |
| G2 | -0.65 (-0.82, -0.48) | <0.001 |  | -0.28 (-0.44, -0.13) | <0.001 |  | -0.03 (-0.12, 0.05) | 0.427 |
| G3 | -1.53 (-1.67, -1.38) | <0.001 |  | -0.65 (-0.80, -0.51) | <0.001 |  | -0.18 (-0.27, -0.09) | <0.001 |
| Prebiotic use |  |  |  |  |  |  |  |  |
| No | 0 [Reference] |  |  | 0 [Reference] |  |  | 0 [Reference] |  |
| Yes | -0.68 (-1.01, -0.35) | <0.001 |  | -0.34 (-0.68, -0.01) | 0.043 |  | -0.07 (-0.26, 0.11) | 0.437 |
| Probiotic use |  |  |  |  |  |  |  |  |
| No | 0 [Reference] |  |  | 0 [Reference] |  |  | 0 [Reference] |  |
| Yes | -1.05 (-1.35, -0.75) | <0.001 |  | -0.59 (-0.91, -0.28) | <0.001 |  | -0.44 (-0.62, -0.27) | <0.001 |

Model 1 was adjusted for age (20-39, 40-59, or ≥60), sex (male or female), race/ethnicity (Non-Hispanic White, Non-Hispanic Black or Other), marital status (married/living with partner, or single/divorced/widowed), education level (below high school, high school, or above high school), family PIR (≤1.0, 1.1–3.0, or >3.0), drinking status (nondrinker, former drinker, or current drinker), BMI (<25.0, 25.0-29.9, or >29.9), physical activity (inactive, insufficiently active, or active), HEI (in quartiles), and Charlson comorbidity index (continuous); Model 2 was adjusted as model 1 plus smoking status (never smoker, former smoker, or current smoker).

† Participants were categorized into three groups based on the MedHi consumption of quantify the ingestion of live microbes: G1, consumers without intakes of any MedHi food; G2, those with intakes of MedHi food above zero but below the median level for consumers; G3, those with intakes of MedHi food above the median level for consumers.
